# PROTACs in Ovarian Cancer: Current Advancements and Future Perspectives

**DOI:** 10.3390/ijms25105067

**Published:** 2024-05-07

**Authors:** Makenzie Vorderbruggen, Carlos A. Velázquez-Martínez, Amarnath Natarajan, Adam R. Karpf

**Affiliations:** 1 Eppley Institute for Research in Cancer, University of Nebraska Medical Center, Omaha, NE 68198-6805, USA; mvorderbruggen@unmc.edu (M.V.); anatarajan@unmc.edu (A.N.); 2 Fred & Pamela Buffett Cancer Center, University of Nebraska Medical Center, Omaha, NE 68198-6805, USA; 3 Faculty of Pharmacy and Pharmaceutical Sciences, University of Alberta, Edmonton, AB T6G 2E1, Canada; velazque@ualberta.ca

**Keywords:** ovarian cancer, PROTAC, targeted protein degradation, high-grade serous ovarian cancer

## Abstract

Ovarian cancer is the deadliest gynecologic malignancy. The majority of patients diagnosed with advanced ovarian cancer will relapse, at which point additional therapies can be administered but, for the most part, these are not curative. As such, a need exists for the development of novel therapeutic options for ovarian cancer patients. Research in the field of targeted protein degradation (TPD) through the use of proteolysis-targeting chimeras (PROTACs) has significantly increased in recent years. The ability of PROTACs to target proteins of interest (POI) for degradation, overcoming limitations such as the incomplete inhibition of POI function and the development of resistance seen with other inhibitors, is of particular interest in cancer research, including ovarian cancer research. This review provides a synopsis of PROTACs tested in ovarian cancer models and highlights PROTACs characterized in other types of cancers with potential high utility in ovarian cancer. Finally, we discuss methods that will help to enable the selective delivery of PROTACs to ovarian cancer and improve the pharmacodynamic properties of these agents.

## 1. Ovarian Cancer

Ovarian cancer is estimated to be the fifth leading cause of cancer-related deaths among U.S. women [1]. Ovarian cancer is broadly subdivided into three types: germ cell, sex-cord stromal, and epithelial (EOC), with EOC being the most common, accounting for approximately 90% of cases [2]. EOC is further subdivided into mucinous, endometrioid, clear cell, low-grade serous (LGSC), and high-grade serous (HGSOC), with HGSOC accounting for 70–80% of EOC cases, making it the most common subtype of ovarian cancer [2].

Treatment of EOC consists of debulking surgery coupled with chemotherapy [2]. When possible, patients receive primary debulking surgery to remove as much of the tumor as is feasible, followed by adjuvant platinum- and taxane-based chemotherapy [3]. In cases of highly advanced disease or difficult-to-resect tumors, interval debulking surgery is used, where patients first receive primary chemotherapy to reduce the disease burden followed by debulking surgery [3]. Approximately 70% of patients treated with platinum- and taxane-based chemotherapy respond favorably to treatment and achieve remission [4]. However, one of the major challenges with ovarian cancer is recurrence, as 70% of patients diagnosed with advanced stage disease (FIGO stage III or IV) that receive chemotherapy develop recurrent disease within 5 years [5]. Following relapse, patients receive additional chemotherapy, the VEGF inhibitor bevacizumab, and/or PARP inhibitors [3]. Despite the success of these new approaches, they are rarely curative; thus, there remains a dire need for the development of novel therapeutic options for patients with EOC, particularly for those with recurrent disease.

## 2. Ubiquitin–Proteasome System

The ubiquitin–proteasome system (UPS) is a key regulator of cellular protein degradation [6]. In the UPS, ubiquitin, a 76 amino acid protein, is conjugated to lysine residues of target proteins by a series of enzymatic reactions [6,7]. Ubiquitin is initially conjugated to E1 ubiquitin-activating enzymes, followed by transfer to E2 ubiquitin-conjugating enzymes, which work in concert with one of four different classes of E3 ligases to transfer ubiquitin to target proteins [7]. The U-box and really interesting new gene (RING) classes of E3 ligases bring together E2-ubiquitin-conjugating enzyme and target proteins, facilitating direct transfer of ubiquitin to the target [7]. In contrast, the homologous to the E6AP carboxyl terminus (HECT) and RING-between-RING (RBR) classes of E3 ligases function through the conjugation of ubiquitin from the E2 ubiquitin-conjugating enzyme to the E3 ligase, followed by transfer to the target protein [7]. In order for target proteins to be degraded through the 26S proteasome, additional ubiquitin proteins must be conjugated through the K48 residue of ubiquitin, forming a polyubiquitin chain [7]. K48 polyubiquitination is recognized by receptors in the 19S regulatory subunits of the 26S proteasome, facilitating the entry of proteins to the 20S core subunit and subsequent cleavage of proteins to peptides through an ATP-dependent process, resulting in target protein degradation [6].

## 3. Proteolysis Targeting Chimeras

**Figure 1:**
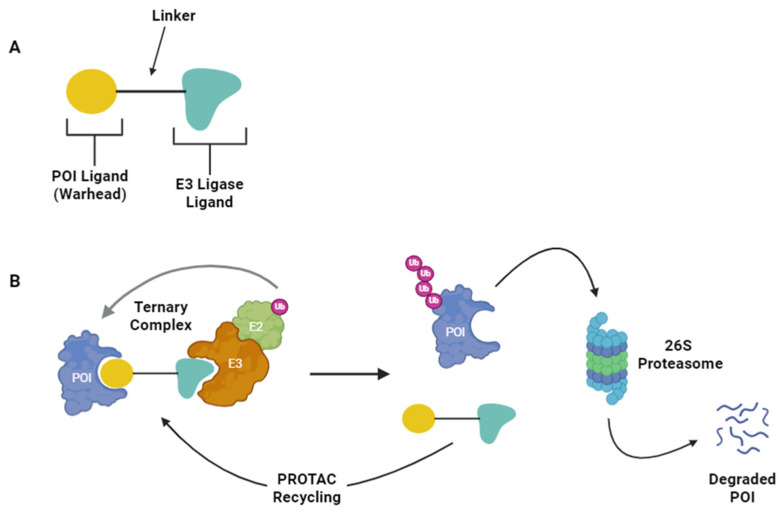
PROTAC structure and mechanism of action (MOA): (**A**) the PROTAC structure includes a warhead that binds the POI, a linker, and an E3 ligase ligand; and (**B**) the PROTAC MOA includes the formation of a ternary complex comprised of the POI, the PROTAC, and the E3 ligase. The transfer of Ubiquitin to the POI leads to its proteolytic degradation by the proteosome, while the PROTAC is recycled and can engage another molecule of POI. Figure created with BioRender.com.

Proteolysis targeting chimeras (PROTACs) take advantage of the UPS to accomplish targeted protein degradation for therapeutic purposes. Structurally, PROTACs are heterobifunctional molecules composed of: (1) a ligand that binds a protein of interest (POI), commonly referred to as the warhead; (2) a linker motif; and (3) an E3-ligase-recruiting ligand (Figure 1) [8]. PROTAC engagement with the POI and an E3 ligase facilitates the formation of a ternary complex, which leads to the polyubiquitination and proteasomal degradation of the POI [8]. The first PROTACs were designed to target POIs including methionine aminopeptidase 2 (MetAP2), the estrogen receptor (ER), and the androgen receptor (AR) [9,10]. The E3-ligase-recruiting moiety of these early PROTACs was peptide-based, using the IκBα phosphopeptide to recruit the SCF^β-TRCP^ E3 ligase [9,10]. The first completely small-molecule-based PROTAC that targeted the AR was composed of a non-steroidal androgen receptor ligand as the warhead and nutllin, a ligand for the E3 ligase mouse double minute-2 (MDM2) [11]. The discovery of additional small-molecule E3 ligase ligands further expanded the small-molecule-based PROTAC repertoire, allowing for recruitment of von Hippel–Lindau (VHL) and cereblon E3 ligases [12,13,14].

While over 600 E3 ligases have been identified, only a handful have been targeted by PROTACs to date. The most common E3 ligases targeted by PROTACs are cereblon, VHL, MDM2, and inhibitor of apoptosis proteins (IAPs) [15]. Consequently, the E3-ligase-recruiting moiety is an important consideration in PROTAC design. For example, mutations in VHL, as seen in some cancers, and the inherent high molecular weight of this class of PROTACs might limit their broad applicability [16]. Conversely, cereblon-recruiting PROTACs have lower molecular weights, allowing for oral bioavailability, but cereblon shows ubiquitous expression in normal and tumor tissues, which may limit the tumor selectivity of this class of PROTACs [16]. Additionally, an experimental limitation of cereblon-targeting PROTACs comes with the fact that mouse cereblon has a point mutation relative to human cereblon [17]. While cereblon ligands like thalidomide retain the ability to bind mouse cereblon, certain neosubstrates of cereblon are unable to bind due to steric hinderance [17,18]. As such, an accurate assessment of cereblon-targeting PROTAC pre-clinical activity may not be possible in syngeneic mouse models and the toxicity profiles of such PROTACs in syngeneic and xenograft models may not accurately recapitulate the effects in humans.

Another important consideration in PROTAC design is the linker. Linker composition can impact PROTAC solubility and cell permeability [19]. While the majority of linkers are composed of alkyl or polyethylene glycol (PEG) chains, additional functional groups are now being incorporated to improve the physicochemical properties of PROTACs [19]. Additionally, an optimal distance between the POI and E3 ligase ligand is required for efficient ubiquitination, as short linkers may cause steric hinderance while long linkers may allow for too much movement between the POI and E3 ligase ligand, both of which can limit ternary complex formation [16,20].

In theory, PROTACs afford several advantages over standard small-molecule inhibitors of proteins. While most inhibitors function in a one-to-one ratio with the POI, PROTACs can instead be recycled after targeting a POI for degradation [8]. Recycling allows for a single PROTAC to target multiple copies of a POI, potentially decreasing the amount of drug required to impair the POI, which may reduce toxicity [8,21]. Thus, PROTACs have a mechanism of action (MOA) reminiscent of enzymes, which has been referred to as a pseudo-catalytic MOA [8]. This MOA may afford PROTACs an improved ability to target previously undruggable proteins such as transcription factors [16]. Furthermore, as compared to classical small-molecule inhibitors (SMIs), PROTACs can show greater selectivity for POIs [16,22]. Additionally, PROTACs might be less sensitive to certain resistance mechanisms observed for traditional SMIs such as mutation or upregulation of the target POI [23].

## 4. PROTACs in Ovarian Cancer

**Figure 2:**
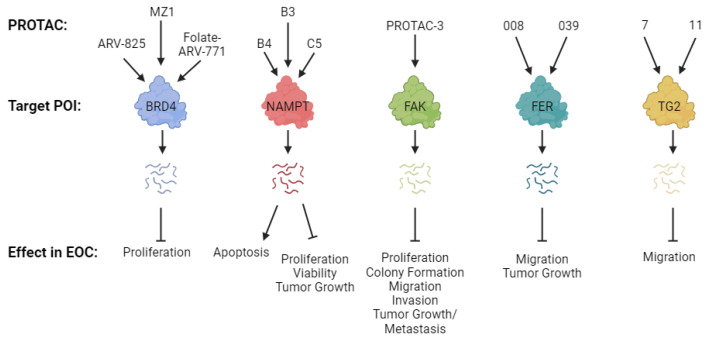
Biological effects of PROTACs tested in EOC models. Figure created with BioRender.com.

To date, several PROTACs have been developed and their effects characterized in EOC models (Table 1, Figure 2). Table 1 provides a breakdown of the components of the PROTACs characterized in EOC, while Figure 2 depicts the biological effects observed with the PROTACs. The most common PROTACs reported using EOC models are described below.

### 4.1. Bromodomain-Containing Protein 4 (BRD4) PROTACs

BRD4, a member of the bromodomain and extra terminal domain (BET) family of proteins, promotes tumorigenesis through its transcriptional co-activator activity and plays roles in processes including DNA damage repair and cellular responses to stress [32]. Given these oncogenic roles, as well as its frequent amplification and overexpression in ovarian cancer, BRD4 is a viable therapeutic target [32,33,34]. To date, multiple BRD4-targeting PROTACs have been developed and characterized in EOC models [24,25]. Nobleja-Lopez and colleagues utilized two previously developed PROTACs, ARV-825 and MZ1, to target BRD4 in EOC [24]. ARV-825 is composed of the BET SMI OTX015 and pomalidomide as the ligand for cereblon, while MZ1 contains a different BET SMI, JQ1, as the warhead that is conjugated to a VHL ligand [35,36]. Nobleja-Lopez and colleagues observed degradation of BRD4 following treatment with ARV-825 or MZ1 in triple-negative breast cancer (TNBC) models, including BET-inhibitor-resistant models, and antiproliferative activity in EOC models [24]. Notably, the BRD4-targeting PROTACs exhibited greater antiproliferative activity than the BET SMIs [24]. Recently, a third BRD4-targeting PROTAC, ARV-771, composed of the BET-binding moiety triazolo-diazepine acetamide and a VHL ligand, resulted in BRD2/3/4 degradation in castration-resistant prostate cancer models [37]. Relevant to EOC, Liu and colleagues further modified ARV-771, creating a folate-caged version [25]. Folate receptor α (FRα) is an important target for drug delivery in EOC, given its increased expression in malignant ovarian cancer relative to benign conditions or the normal ovary, its localization on the plasma membrane, and its ability to internalize large molecules through receptor-mediated endocytosis [38,39]. Consistently, the first FRα-targeted agent was recently FDA-approved for the treatment of recurrent EOC [40]. Notably, folate-ARV-771 resulted in proteasome and folate-dependent BRD4 degradation and selectively diminished proliferation in EOC models over the non-transformed HFF-1, HK2, and 3T3 cell lines [25]. However, the activity of BRD4-targeting PROTACs has yet to be reported in in vivo EOC models.

### 4.2. NAM Phosphoribosyltransferase (NAMPT) PROTACs

NAMPT is a key metabolic regulator involved in the synthesis of nicotinamide adenine dinucleotide (NAD^+^) and is frequently overexpressed in multiple cancers, including EOC [41]. This overexpression is thought to be due to increased rates of proliferation within tumors and, consequently, higher energy demands [42]. While several NAMPT inhibitors have entered clinical trials, their success has been limited by toxicity, necessitating the development of new therapeutic approaches [27]. In response to this challenge, the first NAMPT-targeting PROTAC, PROTAC B4, was developed using the NAMPT inhibitor and fluorescent compound, M049-0244, connected to a VHL ligand [26,43]. Excitation of PROTAC B4 with visible light resulted in fluorescence, the intensity of which increased in the presence of NAMPT, allowing for confirmation that the compound entered cells, resulting in NAMPT binding and its subsequent proteasomal degradation [26]. PROTAC B4 selectively diminished the proliferation of EOC cells relative to noncancerous human umbilical vein endothelial cells. Moreover, intraperitoneal (IP) administration of PROTAC B4 to nude mice harboring A2780 xenografts led to reduced tumor growth without overt toxicity, as evidenced by minimal changes in mouse bodyweight. This was an improvement over the parental compound, M049-0244, which showed moderate antitumor activity with high toxicity, as evidenced by substantial decreases in mouse bodyweight. A second NAMPT-targeting PROTAC, PROTAC B3, was similarly developed, again using a VHL ligand, but incorporating an alternative NAMPT inhibitor, MS0, as the warhead [27]. PROTAC B3 induced the proteasomal degradation of NAMPT and decreased EOC cell viability. The compound also reduced A2780 tumor growth in a subcutaneous xenograft nude mouse model with no overt toxicity, as evidenced by minimal changes in bodyweight and the normal histology of several organs, including the heart, liver, spleen, lung, and kidney [27]. This was an improvement over the parental MS0 compound, which induced toxicity, as evidenced by decreased bodyweight and abnormalities in the kidney tissues. These differences in toxicity profiles between the PROTAC and the parental compound may be due to the improved selectivity of the PROTACs [16,22]. A third NAMPT-targeting PROTAC, PROTAC C5, was recently developed using the NAMPT inhibitor FK866 connected to a VHL ligand [28]. Interestingly, a comparison of various linker lengths demonstrated that PROTAC C5, with an eight-carbon atom linker, resulted in stronger NAMPT degradation as compared to linker lengths of 4–7 or 9–11 carbon atoms. This observation reinforces the notion that linker length and composition is an important aspect that influences PROTAC target engagement [16,19,20]. While PROTAC C5 inhibited EOC proliferation and induced EOC cell apoptosis, this PROTAC has not yet been tested in vivo [28].

Although the NAMPT-targeting PROTACs B3 and B4 both showed activity in vivo, the findings were limited to immunodeficient xenograft models. In contrast, Wu and colleagues developed an NAMPT-targeting PROTAC, PROTAC A7, using the NAMPT inhibitor MS7 connected to a VHL ligand and observed reduced tumor growth following subcutaneous injection of CT26 mouse colorectal cancer cells in the immunocompetent BALB/c mouse model [44]. Importantly, this work showed that NAMPT-targeting PROTACs can affect the immune microenvironment by decreasing myeloid-derived suppressor cell populations and by increasing T cell tumor infiltration. Given these results, it will be relevant for future studies characterizing the activity of NAMPT-targeting PROTACs in EOC to use immunocompetent models in order to assess potential effects on the immune microenvironment.

### 4.3. Focal Adhesion Kinase (FAK) PROTACs

The gene encoding FAK, a non-receptor tyrosine kinase, is frequently amplified in EOC [45]. High FAK mRNA expression is correlated with reduced progression-free and overall survival of EOC patients [29]. FAK plays a role in tumorigenesis by promoting cell proliferation, migration, invasion, and adhesion, pointing to the kinase as a potential therapeutic target in EOC [46]. FAK SMIs have been developed to target its kinase-dependent functions but fail to target kinase-independent functions such as its function as a scaffold protein, mediating protein–protein interactions [29]. This is a limitation that PROTACs are well-suited to address. PROTAC-3 was developed to target FAK using a modified form of the FAK SMI, defacitinib, as the warhead, in combination with a VHL ligand [47]. Treatment of breast cancer cells with this compound resulted in FAK degradation with greater selectivity for FAK over other kinases, as compared with defacitinib, and decreased cell migration and invasion. Huo and colleagues further assessed the activity of PROTAC-3 in EOC cell lines and observed that the compound reduced FAK and p-FAK levels in addition to inducing greater reductions in cell proliferation, colony formation, cell migration, and cell invasion compared with defacitinib [29]. Moreover, PROTAC-3, but not defacitinib, disrupted the interaction of FAK with ASAP1 (ADP-ribosylation factor (ARF) GTPase-activating protein), demonstrating the ability of the compound to successfully inhibit the kinase-independent functions of FAK. Importantly, PROTAC-3 also showed activity against EOC xenografts, decreasing the growth and metastasis of OVCAR8 cells injected intrabursally into female NSG mice. These data suggest that the degradation of FAK is a more effective therapeutic approach than is the inhibition of its kinase activity with SMIs.

### 4.4. Feline Sarcoma-Related Kinase (FER) PROTACs

FER kinase is a potential therapeutic target in EOC, as it is frequently overexpressed and plays a functional role in EOC metastasis [48]. Two FER-targeting PROTACs, SIAIS25008 and SIAIS262039, abbreviated as 008 and 039, respectively, have recently been described [30]. These two compounds were modified from a previously developed PROTAC, SIAIS164018, composed of the cereblon ligand pomalidomide linked to brigatinib C. Brigatinib C is a derivative of the SMI brigatinib, which was originally designed to inhibit anaplastic lymphoma kinase (ALK) and epidermal growth factor receptor (EGFR) [30,49]. Unexpectedly, SIAIS1764018 was observed to promote the degradation of FER in addition to its intended target, ALK [49]. To improve the FER degradation properties, Zhang and colleagues structurally optimized SIAIS1764018, swapping pomalidomide for lenalidomide and altering the linker length and composition, creating PROTACs 008 and 039 [30]. Both of these new PROTACs effectively promoted proteasome-dependent FER degradation in EOC cells [30]. While PROTACs 008 and 039 did not significantly affect EOC cell proliferation, cell-cycle progression, or cell apoptosis, the compounds decreased EOC cell migration, and did so at a lower concentration than brigatinib, consistent with the known role of FER in EOC metastasis [30,48]. Treatment of female NSG mice with PROTAC 008 decreased the tumor burden of CAOV4 IP xenografts [30]. Intriguingly, a greater reduction in tumor xenograft burden was observed when combining PROTAC 008 treatment with FER knockdown, possibly due to the off-target degradative activity of PROTAC 008 on other kinases such as AAK1 and GAK or the PROTAC-mediated inhibition of cereblon.

### 4.5. Transglutaminase 2 (TG2) PROTACs

TG2 is upregulated in EOC, which is associated with reduced overall survival in patients [50,51,52]. In normal physiology, TG2 binds fibronectin when localized to the plasma membrane or secreted into the extracellular matrix (ECM) and thus plays a functional role in cell adhesion and ECM remodeling [53]. TG2 can also localize to the cytosol, where it interacts with GTP and influences intracellular signaling [54,55]. In the context of cancer, these functions can be hijacked, promoting metastasis and tumor dissemination [50,51,54]. While SMIs targeting the TGase activity to disrupt fibronectin binding and SMIs targeting the GTPase activity of TG2 have been developed, their success has been limited, likely due to the multifaceted roles TG2 plays in oncogenesis [31]. Valdivia and colleagues created a series of TG2-targeting PROTACs using the TG2 SMI MT4 connected to either VHL or cereblon ligands [31]. Of the series of compounds synthesized, two VHL-ligand-containing degraders, PROTACs 7 and 11, were found to directly bind TG2, resulting in TG2 proteasomal degradation. Both PROTACs diminished the migration of EOC cells and decreased EOC cell adhesion to fibronectin, without inducing EOC cell death. It is plausible that these compounds may be useful to impair the EOC metastatic burden, but this requires further assessment using in vivo EOC models. Additionally, since neither PROTAC induced cell death, these compounds may also benefit from combination treatments with cytotoxic agents.

## 5. Novel Potential PROTAC Targets in EOC

The development of PROTACs as a treatment strategy for EOC is in its infancy. While a few oncogenic proteins have been successfully targeted by PROTACs in EOC models (Figure 2), there remains great potential for PROTACs characterized in other types of cancer to be developed in EOC. Some of the most prominent examples of these are PROTACs that target Poly ADP Ribose Polymerase 1 (PARP1) [56,57,58,59,60], Forkhead Box M1 (FOXM1) [61,62], c-Myc (MYC) [63], Epidermal Growth Factor Receptor (EGFR) [60,64,65,66,67,68,69,70,71,72,73,74,75,76,77,78,79,80,81,82,83,84], and Cyclin Dependent Kinases (CDK) 2 [85,86,87,88,89] and 9 [22,89,90,91,92,93,94,95,96,97]. We will discuss two of these new classes of PROTACs in detail below, those that target PARP1 and those that target FOXM1.

PARP1 plays a multi-faceted role in DNA damage repair [98]. Most notably, PARP1 stimulates base excision repair (BER) to repair DNA single-strand breaks (SSB) [98]. Importantly, in the presence of PARPi, SSBs accumulate and ultimately lead to DNA double-strand breaks (DSB), which must be repaired to maintain cell viability [99,100]. In homologous recombination (HR)-proficient cells, the resulting DSBs are repaired via the error-free HR mechanism [101]. However, in HR-deficient (HRD) cells, non-homologous end joining (NHEJ), an error-prone repair mechanism, is utilized to repair DSB [101]. This leads to the accumulation of mutations in HRD cells treated with PARP inhibitors (PARPi) and reduced cell viability as compared to HR-proficient cells, a concept referred to as synthetic lethality [102]. Importantly, several PARP SMIs are FDA-approved for use in HRD EOC patients [102]. While PARP inhibitors have shown beneficial activity in the clinic, due to the diverse functions of these enzymes, the field may further benefit from the development of PARP-targeting PROTACs [57].

Two PROTACs, Compound 3 and NN3, were recently developed using the PARPi niraparib as the warhead in combination with a ligand for the MDM2 E3 ligase [56,57]. Compound 3 increased PARP1 cleavage and apoptosis of breast cancer cells. This compound also showed selectivity for breast cancer cells over normal breast cells and further decreased breast cancer cell viability as compared to commonly used PARP inhibitors, including niraparib, olaparib, and veliparib [56]. Similarly, NN3 promoted PARP1 proteasomal degradation in addition to causing the degradation of mutant forms of PARP1 that have been identified in patients and contribute to PARPi resistance [57]. NN3 treatment induced ferroptosis in breast cancer cells and reduced breast cancer xenograft growth to a greater extent than niraparib alone, with minimal changes in mouse bodyweight. Additional PARP1-targeting PROTACs have been developed using a second PARP inhibitor, olaparib, in combination with cereblon ligands [58,59]. One of these PROTACs, Compound 2, induced PARP1 degradation, apoptosis, G1 cell cycle arrest, and reduced proliferation in colorectal cancer cells [58]. However, this PROTAC was limited by a very short half-life (1.86 min) in human liver microsomes. A second olaparib-based PROTAC, LB23, also induced PARP1 degradation in addition to cell-cycle arrest in the G2/M phase in breast cancer cells [59].

Zheng and colleagues developed a novel PROTAC for the dual targeting of EGFR and PARP, given the contribution of both proteins to therapy resistance [60]. These dual PROTACs use a star-type linker composed of either a serine or a tyrosine amino acid, each of which has three reactive sites, allowing for one copy each of the PARP inhibitor, olaparib, the EGFR inhibitor, Gefitinib, and an E3 ligase ligand (either a cereblon or VHL ligand) to be connected by the linker. They reported two such PROTACs, DP-C-1, containing a cereblon ligand and DP-V-4, containing a VHL ligand. Both novel PROTACs induced proteasome-dependent degradation of both EGFR and PARP. DP-V-4 displayed antiproliferative activity in non-small cell lung cancer cells, but to a lesser extent than Gefitinib alone, likely due to reduced solubility and poor cell permeability of the PROTAC. While this affords a unique approach for dual protein-targeting, with the potential to replace certain combination treatments [60], additional work characterizing the anti-cancer activity and pharmacokinetics (PK) of dual-targeting PROTACs is required.

A second potentially high-impact target for PROTACs in EOC is the FOXM1 transcription factor. Dysregulation of the FOXM1 gene and pathway is the second most common molecular alteration in HGSOC (after TP53 mutations), and the upregulation of FOXM1 is associated with the reduced progression-free and overall survival of EOC patients [103,104,105]. Furthermore, FOXM1 is commonly expressed in recurrent chemoresistant EOC and targeting FOXM1 using SMIs shows anti-cancer effects in EOC cells [106,107,108]. Although FOXM1 has been targeted with SMIs, many of these compounds do not fully disrupt the oncogenic activity of FOXM1 as they only target its DNA-binding activity [107,109]. In contrast, FOXM1-targeted PROTACs might have greater anti-tumor activity as they promote protein degradation and thus fully disrupt FOXM1 functions. To this end, Luo and colleagues developed the first FOXM1-targeting PROTAC, 17d, using an analog of the FOXM1 SMI FDI-6 linked to a cereblon ligand [61]. 17d induced FOXM1 degradation and disrupted the FOXM1 pathway in breast cancer cells. This PROTAC also decreased the growth of breast cancer cells with little effect on normal breast cells. Additionally, treatment with 17d promoted apoptosis, G2/M arrest, downregulated epithelial–mesenchymal transition (EMT) genes and reduced the growth of MDA-MB-231 cell xenografts grown subcutaneously in female nude mice.

A second FOXM1-targeting PROTAC, FOXM1-PROTAC was recently reported and consists of a cereblon ligand coupled to FIP-1, a FOXM1-binding peptide identified through a library screen and modified to improve cell permeability [62]. FOXM1 PROTAC resulted in FOXM1 degradation and reduced the viability of breast, lung, colon, and liver cancer cells. FOXM1-PROTAC also decreased migration and colony formation of breast and liver cancer cells. Additionally, FOXM1-PROTAC reduced the growth of subcutaneous HepG2 cell line xenografts in female BALB/c nude mice, and to a greater extent than FIP-1. Mouse bodyweights remained constant following FOXM1-PROTAC treatment and immunohistochemical staining of tissues and measures of liver indexes showed little toxicity. This may however not fully represent the toxicity profile of this PROTAC given the difference between mouse and human cereblon, as previously discussed [17,18].

## 6. Strategies for Targeted Delivery of PROTACs in EOC

**Figure 3:**
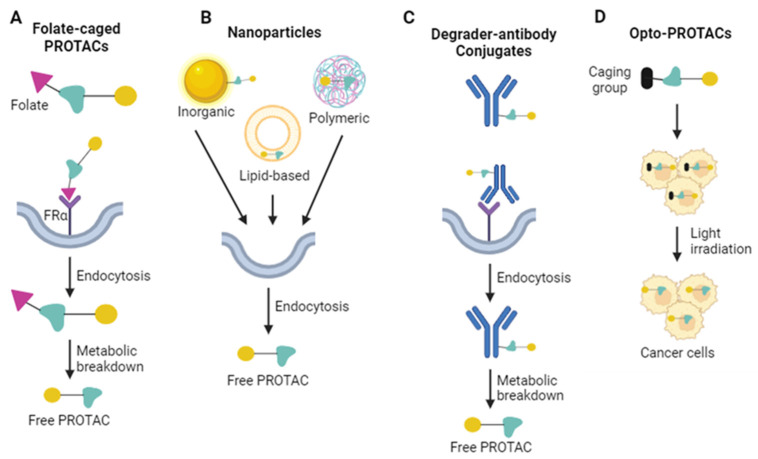
Methods to improve selective delivery of PROTACs in EOC: (**A**) conjugation of folate to PROTACs results in selectivity for cells expressing FRα; (**B**) inorganic, lipid-based, and polymeric nanoparticle-based PROTAC delivery; (**C**) conjugation of PROTACs to antibodies facilitates selective delivery; and (**D**) light irradiation removes the caging group on opto-PROTACs, activating the PROTAC. Created with BioRender.com.

While PROTACs afford several advantages over traditional SMIs, they may have a greater propensity for on-target toxicity in non-target cells due to their degradation of the POI, which leads to near or complete loss of POI downstream functions [110]. As such, methods to improve the selective delivery of PROTACs for tumor cells versus normal cells will be key for the continued progression of these compounds to the clinic. In this context, methods to enable the selective delivery of PROTACs to EOC cells are of paramount importance. To this end, as mentioned above, Liu and colleagues developed a folate-caged PROTAC for the targeted delivery of a BRD4 PROTAC to EOC cells, based on targeting FRα (Figure 3A) [25]. In addition to this approach, the potential exists for the use of additional delivery systems, such as nanoparticles and degrader-antibody conjugates, to improve cell permeability, selectivity, and the pharmacodynamics of PROTACs in ovarian cancer. For example, inorganic, lipid-based, or polymeric nanoparticles can be used to package PROTACs for improved stability and selectivity (Figure 3B) [111]. Such systems have successfully been used to deliver BRD4-targeting PROTACs and have shown anticancer activity in melanoma, pancreatic, and breast cancer models [112,113,114]. Additionally, degrader–antibody conjugates serve as an alternative delivery strategy whereby an antibody with specificity for tumor cells is conjugated to the PROTAC to facilitate targeted delivery (Figure 3C) [111]. For example, antibodies against human epidermal growth factor receptor 2 (HER2), C-type lectin-like molecule-1 (CLL1) and six-transmembrane epithelial antigen of the prostate 1 (STEAP1) have successfully been conjugated to BET-targeting PROTACs [114,115,116,117,118]. The use of nanoparticles and degrader–antibody conjugates for BET-targeting PROTAC delivery is of particular interest given the activity of BRD4 PROTACs in EOC [24,25].

While targeted delivery methods may enhance selectivity, recent advances in the field of opto-PROTACs may afford an alternative method for achieving selectivity through the spatiotemporal control of PROTAC activation (Figure 3D). These strategies use caging groups or photoswitches that serve to maintain the PROTAC in an inactive state until exposed to light [110,119,120,121]. Opto-PROTACs have now been used to successfully target several POIs, including ALK, BRD2, BRD3, BRD4, and BTK [110,119,120,121]. These compounds promoted light-dependent POI degradation and resulted in antiproliferative effects in cancer models including Burkett’s lymphoma, hepatocellular carcinoma, lymphoma, and non-small cell lung cancer. The potential activity of opto-PROTACs in an in vivo setting, however, remains to be characterized. Recent work by Liu and colleagues is of particular interest, as the caging group was added to pomalidomide, which may make the technology more readily applicable to the pomalidomide-based PROTACs currently under investigation in EOC [24,120]. While opto-PROTACs provide a novel strategy for selectively targeting cancer, the technology is currently limited to blood and skin cancers due to the inability of the light used for irradiation to penetrate tissues [120]. Future methods could potentially use caging groups or photoswitches activated in the near-infrared region, allowing for improved tissue penetration, increasing the technology’s applicability across an array of cancers, including EOC [120].

## 7. Conclusions

Several different PROTACs have recently been investigated in EOC models, although the field remains in its infancy. Further research is needed to investigate new POIs that may provide therapeutic benefits in EOC, as well as to better characterize the activity of existing PROTACs targeting POIs in EOC using in vivo EOC models such as xenografts, PDXs, and immunocompetent syngeneic models. An exciting development in the field has been the use of a folate-caged PROTAC for targeted delivery to FRα-expressing cells, which has high relevance for EOC [25,38,39]. Future work focused on additional delivery systems, such as nanoparticles, degrader–antibody conjugates, and opto-PROTACs, will provide further opportunities to refine PROTAC delivery and selectivity for EOC.

## Figures and Tables

**Table 1:** Chemical constituents of PROTACs characterized in EOC models.

PROTAC Name	Target Protein	Warhead	Linker	E3 Ligase Ligand	E3 Ligase	Reference
ARV-825	BRD4	OTX015	PEG	Pomalidomide	Cereblon	[24]
MZ1	BRD4	JQ1	PEG	VHL-1	VHL	[24]
Folate-ARV-771	BRD4	Triazolo-diazepine Acetamide	PEG	VHL-1	VHL	[25]
B4	NAMPT	M049-0244	Alkyl	VHL ligand 1	VHL	[26]
B3	NAMPT	MS0	Alkyl	VHL ligand 9	VHL	[27]
C5	NAMPT	FK866	Alkyl	VHL ligand 7	VHL	[28]
PROTAC-3	FAK	Defacitinib	PEG	VHL-1	VHL	[29]
SIAIS25008 (008)	FER	Brigatinib	Alkyl	Lenalidomide	Cereblon	[30]
SIAIS262039 (039)	FER	Brigatinib	Alkyl	Lenalidomide	Cereblon	[30]
7	TG2	MT4	PEG	VHL-1	VHL	[31]
11	TG2	MT4	PEG	VHL-1	VHL	[31]

## Data Availability

Not applicable.

## References

[1] Siegel R.L., Miller K.D., Wagle N.S., Jemal A. (2023). Cancer statistics, 2023. CA Cancer J. Clin.

[2] Stewart C., Ralyea C., Lockwood S. (2019). Ovarian Cancer: An Integrated Review. Semin. Oncol. Nurs..

[3] Morand S., Devanaboyina M., Staats H., Stanbery L., Nemunaitis J. (2021). Ovarian Cancer Immunotherapy and Personalized Medicine. Int. J. Mol. Sci..

[4] Lisio M.A., Fu L., Goyeneche A., Gao Z.H., Telleria C. (2019). High-Grade Serous Ovarian Cancer: Basic Sciences, Clinical and Therapeutic Standpoints. Int. J. Mol. Sci..

[5] Kurnit K.C., Fleming G.F., Lengyel E. (2021). Updates and New Options in Advanced Epithelial Ovarian Cancer Treatment. Obstet. Gynecol..

[6] Park J., Cho J., Song E.J. (2020). Ubiquitin-proteasome system (UPS) as a target for anticancer treatment. Arch. Pharm. Res..

[7] Yang Q., Zhao J., Chen D., Wang Y. (2021). E3 ubiquitin ligases: Styles, structures and functions. Mol. Biomed..

[8] Békés M., Langley D.R., Crews C.M. (2022). PROTAC targeted protein degraders: The past is prologue. Nat. Rev. Drug Discov..

[9] Sakamoto K.M., Kim K.B., Kumagai A., Mercurio F., Crews C.M., Deshaies R.J. (2001). Protacs: Chimeric molecules that target proteins to the Skp1-Cullin-F box complex for ubiquitination and degradation. Proc. Natl. Acad. Sci. USA.

[10] Sakamoto K.M., Kim K.B., Verma R., Ransick A., Stein B., Crews C.M., Deshaies R.J. (2003). Development of Protacs to target cancer-promoting proteins for ubiquitination and degradation. Mol. Cell. Proteomics.

[11] Schneekloth A.R., Pucheault M., Tae H.S., Crews C.M. (2008). Targeted intracellular protein degradation induced by a small molecule: En route to chemical proteomics. Bioorg. Med. Chem. Lett..

[12] Buckley D.L., Gustafson J.L., Van Molle I., Roth A.G., Tae H.S., Gareiss P.C., Jorgensen W.L., Ciulli A., Crews C.M. (2012). Small-molecule inhibitors of the interaction between the E3 ligase VHL and HIF1α. Angew. Chem. Int. Ed. Engl..

[13] Buckley D.L., Van Molle I., Gareiss P.C., Tae H.S., Michel J., Noblin D.J., Jorgensen W.L., Ciulli A., Crews C.M. (2012). Targeting the von Hippel-Lindau E3 ubiquitin ligase using small molecules to disrupt the VHL/HIF-1α interaction. J. Am. Chem. Soc..

[14] Ito T., Ando H., Suzuki T., Ogura T., Hotta K., Imamura Y., Yamaguchi Y., Handa H. (2010). Identification of a primary target of thalidomide teratogenicity. Science.

[15] Li J.W., Zheng G., Kaye F.J., Wu L. (2023). PROTAC therapy as a new targeted therapy for lung cancer. Mol. Ther..

[16] Khan S., He Y., Zhang X., Yuan Y., Pu S., Kong Q., Zheng G., Zhou D. (2020). PROteolysis TArgeting Chimeras (PROTACs) as emerging anticancer therapeutics. Oncogene.

[17] Fink E.C., McConkey M., Adams D.N., Haldar S.D., Kennedy J.A., Guirguis A.A., Udeshi N.D., Mani D.R., Chen M., Liddicoat B. (2018). Crbn (I391V) is sufficient to confer in vivo sensitivity to thalidomide and its derivatives in mice. Blood.

[18] Yamamoto J., Ito T., Yamaguchi Y., Handa H. (2022). Discovery of CRBN as a target of thalidomide: A breakthrough for progress in the development of protein degraders. Chem. Soc. Rev..

[19] Troup R.I., Fallan C., Baud M.G.J. (2020). Current strategies for the design of PROTAC linkers: A critical review. Explor. Target. Antitumor Ther..

[20] Cyrus K., Wehenkel M., Choi E.Y., Han H.J., Lee H., Swanson H., Kim K.B. (2011). Impact of linker length on the activity of PROTACs. Mol. Biosyst..

[21] Liu Z., Hu M., Yang Y., Du C., Zhou H., Liu C., Chen Y., Fan L., Ma H., Gong Y. (2022). An overview of PROTACs: A promising drug discovery paradigm. Mol. Biomed..

[22] Robb C.M., Contreras J.I., Kour S., Taylor M.A., Abid M., Sonawane Y.A., Zahid M., Murry D.J., Natarajan A., Rana S. (2017). Chemically induced degradation of CDK9 by a proteolysis targeting chimera (PROTAC). Chem. Commun..

[23] Burke M.R., Smith A.R., Zheng G. (2022). Overcoming Cancer Drug Resistance Utilizing PROTAC Technology. Front. Cell Dev. Biol..

[24] Noblejas-López M.D.M., Nieto-Jimenez C., Burgos M., Gómez-Juárez M., Montero J.C., Esparís-Ogando A., Pandiella A., Galán-Moya E.M., Ocaña A. (2019). Activity of BET-proteolysis targeting chimeric (PROTAC) compounds in triple negative breast cancer. J. Exp. Clin. Cancer Res..

[25] Liu J., Chen H., Liu Y., Shen Y., Meng F., Kaniskan H., Jin J., Wei W. (2021). Cancer Selective Target Degradation by Folate-Caged PROTACs. J. Am. Chem. Soc..

[26] Cheng J., He S., Xu J., Huang M., Dong G., Sheng C. (2022). Making Protein Degradation Visible: Discovery of Theranostic PROTACs for Detecting and Degrading NAMPT. J. Med. Chem..

[27] Bi K., Cheng J., He S., Fang Y., Huang M., Sheng C., Dong G. (2023). Discovery of Highly Potent Nicotinamide Phosphoribosyltransferase Degraders for Efficient Treatment of Ovarian Cancer. J. Med. Chem..

[28] Zhang P., Wang W., Guo M., Zhou L., Dong G., Xu D., Sheng C. (2023). Discovery of potent NAMPT-Targeting PROTACs using FK866 as the warhead. Bioorg. Med. Chem. Lett..

[29] Huo X., Zhang W., Zhao G., Chen Z., Dong P., Watari H., Narayanan R., Tillmanns T.D., Pfeffer L.M., Yue J. (2022). FAK PROTAC Inhibits Ovarian Tumor Growth and Metastasis by Disrupting Kinase Dependent and Independent Pathways. Front. Oncol..

[30] Zhang Y., Xiong X., Sun R., Zhu X., Wang C., Jiang B., Yang X., Li D., Fan G. (2023). Development of the nonreceptor tyrosine kinase FER-targeting PROTACs as a potential strategy for antagonizing ovarian cancer cell motility and invasiveness. J. Biol. Chem..

[31] Valdivia A., Vagadia P.P., Guo G., O’Brien E., Matei D., Schiltz G.E. (2023). Discovery and Characterization of PROTACs Targeting Tissue Transglutaminase (TG2). J. Med. Chem..

[32] Drumond-Bock A.L., Bieniasz M. (2021). The role of distinct BRD4 isoforms and their contribution to high-grade serous ovarian carcinoma pathogenesis. Mol. Cancer.

[33] Goundiam O., Gestraud P., Popova T., De la Motte Rouge T., Fourchotte V., Gentien D., Hupé P., Becette V., Houdayer C., Roman-Roman S. (2015). Histo-genomic stratification reveals the frequent amplification/overexpression of CCNE1 and BRD4 genes in non-BRCAness high grade ovarian carcinoma. Int. J. Cancer.

[34] Petersen S., Wilson A.J., Hirst J., Roby K.F., Fadare O., Crispens M.A., Beeghly-Fadiel A., Khabele D. (2020). CCNE1 and BRD4 co-amplification in high-grade serous ovarian cancer is associated with poor clinical outcomes. Gynecol. Oncol..

[35] Lu J., Qian Y., Altieri M., Dong H., Wang J., Raina K., Hines J., Winkler J.D., Crew A.P., Coleman K. (2015). Hijacking the E3 Ubiquitin Ligase Cereblon to Efficiently Target BRD4. Chem. Biol..

[36] Zengerle M., Chan K.H., Ciulli A. (2015). Selective Small Molecule Induced Degradation of the BET Bromodomain Protein BRD4. ACS Chem. Biol..

[37] Raina K., Lu J., Qian Y., Altieri M., Gordon D., Rossi A.M., Wang J., Chen X., Dong H., Siu K. (2016). PROTAC-induced BET protein degradation as a therapy for castration-resistant prostate cancer. Proc. Natl. Acad. Sci. USA.

[38] Huang M.J., Zhang W., Wang Q., Yang Z.J., Liao S.B., Li L. (2018). FOLR1 increases sensitivity to cisplatin treatment in ovarian cancer cells. J. Ovarian Res..

[39] Mai J., Wu L., Yang L., Sun T., Liu X., Yin R., Jiang Y., Li J., Li Q. (2023). Therapeutic strategies targeting folate receptor α for ovarian cancer. Front. Immunol..

[40] Dilawari A., Shah M., Ison G., Gittleman H., Fiero M.H., Shah A., Hamed S.S., Qiu J., Yu J., Manheng W. (2023). FDA Approval Summary: Mirvetuximab Soravtansine-Gynx for FRα-Positive, Platinum-Resistant Ovarian Cancer. Clin. Cancer Res..

[41] Shackelford R.E., Bui M.M., Coppola D., Hakam A. (2010). Over-expression of nicotinamide phosphoribosyltransferase in ovarian cancers. Int. J. Clin. Exp. Pathol..

[42] Yaku K., Okabe K., Hikosaka K., Nakagawa T. (2018). NAD Metabolism in Cancer Therapeutics. Front. Oncol..

[43] Wang X., Xu T.Y., Liu X.Z., Zhang S.L., Wang P., Li Z.Y., Guan Y.F., Wang S.N., Dong G.Q., Zhuo S. (2015). Discovery of Novel Inhibitors and Fluorescent Probe Targeting NAMPT. Sci. Rep..

[44] Wu Y., Pu C., Fu Y., Dong G., Huang M., Sheng C. (2022). NAMPT-targeting PROTAC promotes antitumor immunity via suppressing myeloid-derived suppressor cell expansion. Acta Pharm. Sin. B.

[45] Kaveh F., Baumbusch L.O., Nebdal D., Børresen-Dale A.L., Lingjærde O.C., Edvardsen H., Kristensen V.N., Solvang H.K. (2016). A systematic comparison of copy number alterations in four types of female cancer. BMC Cancer.

[46] Levy A., Alhazzani K., Dondapati P., Alaseem A., Cheema K., Thallapureddy K., Kaur P., Alobid S., Rathinavelu A. (2019). Focal Adhesion Kinase in Ovarian Cancer: A Potential Therapeutic Target for Platinum and Taxane-Resistant Tumors. Curr. Cancer Drug Targets.

[47] Cromm P.M., Samarasinghe K.T.G., Hines J., Crews C.M. (2018). Addressing Kinase-Independent Functions of Fak via PROTAC-Mediated Degradation. J. Am. Chem. Soc..

[48] Fan G., Zhang S., Gao Y., Greer P.A., Tonks N.K. (2016). HGF-independent regulation of MET and GAB1 by nonreceptor tyrosine kinase FER potentiates metastasis in ovarian cancer. Genes Dev..

[49] Ren C., Sun N., Liu H., Kong Y., Sun R., Qiu X., Chen J., Li Y., Zhang J., Zhou Y. (2021). Discovery of a Brigatinib Degrader SIAIS164018 with Destroying Metastasis-Related Oncoproteins and a Reshuffling Kinome Profile. J. Med. Chem..

[50] Satpathy M., Cao L., Pincheira R., Emerson R., Bigsby R., Nakshatri H., Matei D. (2007). Enhanced peritoneal ovarian tumor dissemination by tissue transglutaminase. Cancer Res..

[51] Shao M., Cao L., Shen C., Satpathy M., Chelladurai B., Bigsby R.M., Nakshatri H., Matei D. (2009). Epithelial-to-mesenchymal transition and ovarian tumor progression induced by tissue transglutaminase. Cancer Res..

[52] Hwang J.Y., Mangala L.S., Fok J.Y., Lin Y.G., Merritt W.M., Spannuth W.A., Nick A.M., Fiterman D.J., Vivas-Mejia P.E., Deavers M.T. (2008). Clinical and biological significance of tissue transglutaminase in ovarian carcinoma. Cancer Res..

[53] Wang Z., Griffin M. (2012). TG2, a novel extracellular protein with multiple functions. Amino Acids.

[54] Fesus L., Piacentini M. (2002). Transglutaminase 2: An enigmatic enzyme with diverse functions. Trends Biochem. Sci..

[55] Facchiano F., Facchiano A., Facchiano A.M. (2006). The role of transglutaminase-2 and its substrates in human diseases. Front. Biosci..

[56] Zhao Q., Lan T., Su S., Rao Y. (2019). Induction of apoptosis in MDA-MB-231 breast cancer cells by a PARP1-targeting PROTAC small molecule. Chem. Commun..

[57] Li G., Lin S.S., Yu Z.L., Wu X.H., Liu J.W., Tu G.H., Liu Q.Y., Tang Y.L., Jiang Q.N., Xu J.H. (2022). A PARP1 PROTAC as a novel strategy against PARP inhibitor resistance via promotion of ferroptosis in p53-positive breast cancer. Biochem. Pharmacol..

[58] Zhang Z., Chang X., Zhang C., Zeng S., Liang M., Ma Z., Wang Z., Huang W., Shen Z. (2020). Identification of probe-quality degraders for Poly(ADP-ribose) polymerase-1 (PARP-1). J. Enzyme Inhib. Med. Chem..

[59] Pu C., Wang S., Luo D., Liu Y., Ma X., Zhang H., Yu S., Lan S., Huang Q., Deng R. (2022). Synthesis and biological evaluation of a tumor-selective degrader of PARP1. Bioorg. Med. Chem..

[60] Zheng M., Huo J., Gu X., Wang Y., Wu C., Zhang Q., Wang W., Liu Y., Liu Y., Zhou X. (2021). Rational Design and Synthesis of Novel Dual PROTACs for Simultaneous Degradation of EGFR and PARP. J. Med. Chem..

[61] Luo G., Lin X., Vega-Medina A., Xiao M., Li G., Wei H., Velázquez-Martínez C.A., Xiang H. (2021). Targeting of the FOXM1 Oncoprotein by E3 Ligase-Assisted Degradation. J. Med. Chem..

[62] Wang K., Dai X., Yu A., Feng C., Liu K., Huang L. (2022). Peptide-based PROTAC degrader of FOXM1 suppresses cancer and decreases GLUT1 and PD-L1 expression. J. Exp. Clin. Cancer Res..

[63] Li X., Zhang Z., Gao F., Ma Y., Wei D., Lu Z., Chen S., Wang M., Wang Y., Xu K. (2023). c-Myc-Targeting PROTAC Based on a TNA-DNA Bivalent Binder for Combination Therapy of Triple-Negative Breast Cancer. J. Am. Chem. Soc..

[64] Du Y., Chen Y., Wang Y., Chen J., Lu X., Zhang L., Li Y., Wang Z., Ye G., Zhang G. (2022). HJM-561, a Potent, Selective, and Orally Bioavailable EGFR PROTAC that Overcomes Osimertinib-Resistant EGFR Triple Mutations. Mol. Cancer Ther..

[65] Gramespacher J.A., Cotton A.D., Burroughs P.W.W., Seiple I.B., Wells J.A. (2022). Roadmap for Optimizing and Broadening Antibody-Based PROTACs for Degradation of Cell Surface Proteins. ACS Chem. Biol..

[66] Zhang W., Li P., Sun S., Jia C., Yang N., Zhuang X., Zheng Z., Li S. (2022). Discovery of highly potent and selective CRBN-recruiting EGFR(L858R/T790M) degraders in vivo. Eur. J. Med. Chem..

[67] Zhang H., Xie R., Ai-Furas H., Li Y., Wu Q., Li J., Xu F., Xu T. (2022). Design, Synthesis, and Biological Evaluation of Novel EGFR PROTACs Targeting Del19/T790M/C797S Mutation. ACS Med. Chem. Lett..

[68] Li Q., Guo Q., Wang S., Wan S., Li Z., Zhang J., Wu X. (2022). Design and synthesis of proteolysis targeting chimeras (PROTACs) as an EGFR degrader based on CO-1686. Eur. J. Med. Chem..

[69] Zhang X., Xu F., Tong L., Zhang T., Xie H., Lu X., Ren X., Ding K. (2020). Design and synthesis of selective degraders of EGFR(L858R/T790M) mutant. Eur. J. Med. Chem..

[70] Cheng M., Yu X., Lu K., Xie L., Wang L., Meng F., Han X., Chen X., Liu J., Xiong Y. (2020). Discovery of Potent and Selective Epidermal Growth Factor Receptor (EGFR) Bifunctional Small-Molecule Degraders. J. Med. Chem..

[71] Aboelez M.O., Belal A., Xiang G., Ma X. (2022). Design, synthesis, and molecular docking studies of novel pomalidomide-based PROTACs as potential anti-cancer agents targeting EGFR(WT) and EGFR(T790M). J. Enzyme Inhib. Med. Chem..

[72] Qu X., Liu H., Song X., Sun N., Zhong H., Qiu X., Yang X., Jiang B. (2021). Effective degradation of EGFR(L858R+T790M) mutant proteins by CRBN-based PROTACs through both proteosome and autophagy/lysosome degradation systems. Eur. J. Med. Chem..

[73] Zhang H., Zhao H.Y., Xi X.X., Liu Y.J., Xin M., Mao S., Zhang J.J., Lu A.X., Zhang S.Q. (2020). Discovery of potent epidermal growth factor receptor (EGFR) degraders by proteolysis targeting chimera (PROTAC). Eur. J. Med. Chem..

[74] Yu X., Cheng M., Lu K., Shen Y., Zhong Y., Liu J., Xiong Y., Jin J. (2022). Exploring Degradation of Mutant and Wild-Type Epidermal Growth Factor Receptors Induced by Proteolysis-Targeting Chimeras. J. Med. Chem..

[75] Rosenberg S.C., Shanahan F., Yamazoe S., Kschonsak M., Zeng Y.J., Lee J., Plise E., Yen I., Rose C.M., Quinn J.G. (2023). Ternary complex dissociation kinetics contribute to mutant-selective EGFR degradation. Cell Chem. Biol..

[76] Shi S., Du Y., Huang L., Cui J., Niu J., Xu Y., Zhu Q. (2022). Discovery of novel potent covalent inhibitor-based EGFR degrader with excellent in vivo efficacy. Bioorg. Chem..

[77] Zhang N.Y., Hou D.Y., Hu X.J., Liang J.X., Wang M.D., Song Z.Z., Yi L., Wang Z.J., An H.W., Xu W. (2023). Nano Proteolysis Targeting Chimeras (PROTACs) with Anti-Hook Effect for Tumor Therapy. Angew. Chem. Int. Ed. Engl..

[78] He K., Zhang Z., Wang W., Zheng X., Wang X., Zhang X. (2020). Discovery and biological evaluation of proteolysis targeting chimeras (PROTACs) as an EGFR degraders based on osimertinib and lenalidomide. Bioorg. Med. Chem. Lett..

[79] Wang K., Zhou H. (2021). Proteolysis targeting chimera (PROTAC) for epidermal growth factor receptor enhances anti-tumor immunity in non-small cell lung cancer. Drug Dev. Res..

[80] Vartak R., Deore B., Sanhueza C.A., Patel K. (2023). Cetuximab-based PROteolysis targeting chimera for effectual downregulation of NSCLC with varied EGFR mutations. Int. J. Biol. Macromol..

[81] Zhao H.Y., Yang X.Y., Lei H., Xi X.X., Lu S.M., Zhang J.J., Xin M., Zhang S.Q. (2020). Discovery of potent small molecule PROTACs targeting mutant EGFR. Eur. J. Med. Chem..

[82] Zhao H.Y., Wang H.P., Mao Y.Z., Zhang H., Xin M., Xi X.X., Lei H., Mao S., Li D.H., Zhang S.Q. (2022). Discovery of Potent PROTACs Targeting EGFR Mutants through the Optimization of Covalent EGFR Ligands. J. Med. Chem..

[83] Cheng W., Li S., Wen X., Han S., Wang S., Wei H., Song Z., Wang Y., Tian X., Zhang X. (2021). Development of hypoxia-activated PROTAC exerting a more potent effect in tumor hypoxia than in normoxia. Chem. Commun..

[84] Cheng W., Li S., Han S., Miao R., Wang S., Liu C., Wei H., Tian X., Zhang X. (2023). Design, synthesis and biological evaluation of the tumor hypoxia-activated PROTACs bearing caged CRBN E3 ligase ligands. Bioorg. Med. Chem..

[85] Hati S., Zallocchi M., Hazlitt R., Li Y., Vijayakumar S., Min J., Rankovic Z., Lovas S., Zuo J. (2021). AZD5438-PROTAC: A selective CDK2 degrader that protects against cisplatin- and noise-induced hearing loss. Eur. J. Med. Chem..

[86] Riching K.M., Schwinn M.K., Vasta J.D., Robers M.B., Machleidt T., Urh M., Daniels D.L. (2021). CDK Family PROTAC Profiling Reveals Distinct Kinetic Responses and Cell Cycle-Dependent Degradation of CDK2. SLAS Discov..

[87] Wang L., Shao X., Zhong T., Wu Y., Xu A., Sun X., Gao H., Liu Y., Lan T., Tong Y. (2021). Discovery of a first-in-class CDK2 selective degrader for AML differentiation therapy. Nat. Chem. Biol..

[88] Kumarasamy V., Gao Z., Zhao B., Jiang B., Rubin S.M., Burgess K., Witkiewicz A.K., Knudsen E.S. (2023). PROTAC-mediated CDK degradation differentially impacts cancer cell cycles due to heterogeneity in kinase dependencies. Br. J. Cancer.

[89] Zhou F., Chen L., Cao C., Yu J., Luo X., Zhou P., Zhao L., Du W., Cheng J., Xie Y. (2020). Development of selective mono or dual PROTAC degrader probe of CDK isoforms. Eur. J. Med. Chem..

[90] Řezníčková E., Krajčovičová S., Peřina M., Kovalová M., Soural M., Kryštof V. (2022). Modulation of FLT3-ITD and CDK9 in acute myeloid leukaemia cells by novel proteolysis targeting chimera (PROTAC). Eur. J. Med. Chem..

[91] Pei J., Xiao Y., Liu X., Hu W., Sobh A., Yuan Y., Zhou S., Hua N., Mackintosh S.G., Zhang X. (2023). Piperlongumine conjugates induce targeted protein degradation. Cell Chem. Biol..

[92] Noblejas-López M.D.M., Gandullo-Sánchez L., Galán-Moya E.M., López-Rosa R., Tébar-García D., Nieto-Jiménez C., Gómez-Juárez M., Burgos M., Pandiella A., Ocaña A. (2022). Antitumoral Activity of a CDK9 PROTAC Compound in HER2-Positive Breast Cancer. Int. J. Mol. Sci..

[93] King H.M., Rana S., Kubica S.P., Mallareddy J.R., Kizhake S., Ezell E.L., Zahid M., Naldrett M.J., Alvarez S., Law H.C. (2021). Aminopyrazole based CDK9 PROTAC sensitizes pancreatic cancer cells to venetoclax. Bioorg. Med. Chem. Lett..

[94] Bian J., Ren J., Li Y., Wang J., Xu X., Feng Y., Tang H., Wang Y., Li Z. (2018). Discovery of Wogonin-based PROTACs against CDK9 and capable of achieving antitumor activity. Bioorg. Chem..

[95] Wu T., Zhang Z., Gong G., Du Z., Xu Y., Yu S., Ma F., Zhang X., Wang Y., Chen H. (2023). Discovery of novel flavonoid-based CDK9 degraders for prostate cancer treatment via a PROTAC strategy. Eur. J. Med. Chem..

[96] Qiu X., Li Y., Yu B., Ren J., Huang H., Wang M., Ding H., Li Z., Wang J., Bian J. (2021). Discovery of selective CDK9 degraders with enhancing antiproliferative activity through PROTAC conversion. Eur. J. Med. Chem..

[97] Tokarski R.J., Sharpe C.M., Huntsman A.C., Mize B.K., Ayinde O.R., Stahl E.H., Lerma J.R., Reed A., Carmichael B., Muthusamy N. (2023). Bifunctional degraders of cyclin dependent kinase 9 (CDK9): Probing the relationship between linker length, properties, and selective protein degradation. Eur. J. Med. Chem..

[98] Ray Chaudhuri A., Nussenzweig A. (2017). The multifaceted roles of PARP1 in DNA repair and chromatin remodelling. Nat. Rev. Mol. Cell Biol..

[99] Lheureux S., Braunstein M., Oza A.M. (2019). Epithelial ovarian cancer: Evolution of management in the era of precision medicine. CA Cancer J. Clin..

[100] Li J., Sun H., Huang Y., Wang Y., Liu Y., Chen X. (2019). Pathways and assays for DNA double-strand break repair by homologous recombination. Acta Biochim. Biophys. Sin..

[101] Ceccaldi R., Rondinelli B., D’Andrea A.D. (2016). Repair Pathway Choices and Consequences at the Double-Strand Break. Trends Cell Biol..

[102] Jiang X., Li X., Li W., Bai H., Zhang Z. (2019). PARP inhibitors in ovarian cancer: Sensitivity prediction and resistance mechanisms. J. Cell. Mol. Med..

[103] Cancer Genome Atlas Research Network (2011). Integrated genomic analyses of ovarian carcinoma. Nature.

[104] Wen N., Wang Y., Wen L., Zhao S.H., Ai Z.H., Wang Y., Wu B., Lu H.X., Yang H., Liu W.C. (2014). Overexpression of FOXM1 predicts poor prognosis and promotes cancer cell proliferation, migration and invasion in epithelial ovarian cancer. J. Transl. Med..

[105] Barger C.J., Zhang W., Hillman J., Stablewski A.B., Higgins M.J., Vanderhyden B.C., Odunsi K., Karpf A.R. (2015). Genetic determinants of FOXM1 overexpression in epithelial ovarian cancer and functional contribution to cell cycle progression. Oncotarget.

[106] Barger C.J., Chee L., Albahrani M., Munoz-Trujillo C., Boghean L., Branick C., Odunsi K., Drapkin R., Zou L., Karpf A.R. (2021). Co-regulation and function of FOXM1/RHNO1 bidirectional genes in cancer. Elife.

[107] Liu C., Barger C.J., Karpf A.R. (2021). FOXM1: A Multifunctional Oncoprotein and Emerging Therapeutic Target in Ovarian Cancer. Cancers.

[108] Liu C., Vorderbruggen M., Muñoz Trujillo C., Kim S.H., Katzenellenbogen J.A., Katzenellenbogen B.S., Karpf A.R. (2024). NB compounds are potent and efficacious FOXM1 inhibitors in high-grade serous ovarian cancer cells. J. Ovarian Res..

[109] Gormally M.V., Dexheimer T.S., Marsico G., Sanders D.A., Lowe C., Matak-Vinković D., Michael S., Jadhav A., Rai G., Maloney D.J. (2014). Suppression of the FOXM1 transcriptional programme via novel small molecule inhibition. Nat. Commun..

[110] Reynders M., Matsuura B.S., Bérouti M., Simoneschi D., Marzio A., Pagano M., Trauner D. (2020). PHOTACs enable optical control of protein degradation. Sci. Adv..

[111] Chen Y., Tandon I., Heelan W., Wang Y., Tang W., Hu Q. (2022). Proteolysis-targeting chimera (PROTAC) delivery system: Advancing protein degraders towards clinical translation. Chem. Soc. Rev..

[112] Fu Y., Rathod D., Patel K. (2020). Protein kinase C inhibitor anchored BRD4 PROTAC PEGylated nanoliposomes for the treatment of vemurafenib-resistant melanoma. Exp. Cell Res..

[113] Saraswat A., Patki M., Fu Y., Barot S., Dukhande V.V., Patel K. (2020). Nanoformulation of PROteolysis TArgeting Chimera targeting ‘undruggable’ c-Myc for the treatment of pancreatic cancer. Nanomedicine.

[114] Cimas F.J., Niza E., Juan A., Noblejas-López M.D.M., Bravo I., Lara-Sanchez A., Alonso-Moreno C., Ocaña A. (2020). Controlled Delivery of BET-PROTACs: In Vitro Evaluation of MZ1-Loaded Polymeric Antibody Conjugated Nanoparticles in Breast Cancer. Pharmaceutics.

[115] Maneiro M.A., Forte N., Shchepinova M.M., Kounde C.S., Chudasama V., Baker J.R., Tate E.W. (2020). Antibody-PROTAC Conjugates Enable HER2-Dependent Targeted Protein Degradation of BRD4. ACS Chem. Biol..

[116] Pillow T.H., Adhikari P., Blake R.A., Chen J., Del Rosario G., Deshmukh G., Figueroa I., Gascoigne K.E., Kamath A.V., Kaufman S. (2020). Antibody Conjugation of a Chimeric BET Degrader Enables in vivo Activity. ChemMedChem.

[117] Dragovich P.S., Pillow T.H., Blake R.A., Sadowsky J.D., Adaligil E., Adhikari P., Bhakta S., Blaquiere N., Chen J., Dela Cruz-Chuh J. (2021). Antibody-Mediated Delivery of Chimeric BRD4 Degraders. Part 1: Exploration of Antibody Linker, Payload Loading, and Payload Molecular Properties. J. Med. Chem..

[118] Dragovich P.S., Pillow T.H., Blake R.A., Sadowsky J.D., Adaligil E., Adhikari P., Chen J., Corr N., Dela Cruz-Chuh J., Del Rosario G. (2021). Antibody-Mediated Delivery of Chimeric BRD4 Degraders. Part 2: Improvement of In Vitro Antiproliferation Activity and In Vivo Antitumor Efficacy. J. Med. Chem..

[119] Xue G., Wang K., Zhou D., Zhong H., Pan Z. (2019). Light-Induced Protein Degradation with Photocaged PROTACs. J. Am. Chem. Soc..

[120] Liu J., Chen H., Ma L., He Z., Wang D., Liu Y., Lin Q., Zhang T., Gray N., Kaniskan H. (2020). Light-induced control of protein destruction by opto-PROTAC. Sci. Adv..

[121] Pfaff P., Samarasinghe K.T.G., Crews C.M., Carreira E.M. (2019). Reversible Spatiotemporal Control of Induced Protein Degradation by Bistable PhotoPROTACs. ACS Cent. Sci..

